# Phlébolithes révélant des malformations veineuses multiples de la jambe: à propos d'un cas et revue de la littérature

**DOI:** 10.11604/pamj.2013.15.99.1908

**Published:** 2013-07-13

**Authors:** Hicham Yacoubi

**Affiliations:** 1 Service d'orthopédie, Hôpital AlFarabi, Oujda, Maroc

**Keywords:** Phlébolithes, malformation veineuse, phleboliths, venous malformation

## Abstract

Les auteurs rapportent le cas d'une jeune patiente de 27 ans, opérée dans l'enfance pour pied bot varus équin gauche, qui consulte dans notre formation pour syndrome douloureux aigu de la jambe gauche, sans notion de traumatisme. Le bilan radiographique standard a révélé de multiples phlébolithes disséminées dans les parties molles de la jambe, révélant des malformations veineuses profondes, confirmées par angioscanner et IRM. Une biopsie exérèse d'une masse à la face interne du 13 supérieur a confirmé le diagnostic histologique de malformation veineuse avec ablation de la calcification et d'un thrombus.

## Introduction

Les malformations veineuses (MV) sont des dysembryogénies du système vasculaire veineux. Elles peuvent envahir tous les organes. Elles se présentent lorsqu'elles sont superficielles sous forme de masses bleutées compressibles à la palpation. Des phlébolithes sont fréquemment présents. Sa symptomatologie est fonction de sa localisation et de sa taille. Le plus souvent sporadique et isolée, la MV peut être associée à d′autres malformations. Les auteurs rapportent un cas de malformation veineuse de la jambe révélé par des phlébolithes et un syndrome douloureux aigu de la jambe.

## Patient et observation

**Figure 1:**
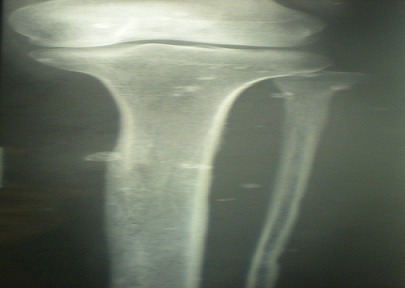
Radiographies standards de la jambe montrant des phlébolithes stigmates d'une malformation veineuses: Vue 1

**Figure 2:**
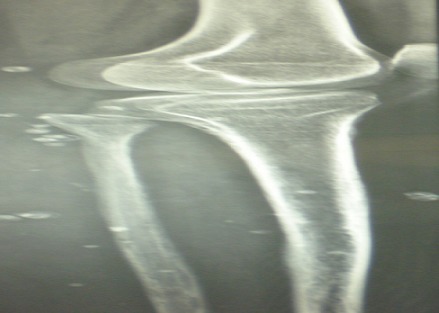
Radiographies standards de la jambe montrant des phlébolithes stigmates d'une malformation veineuses: Vue 2

**Figure 3:**
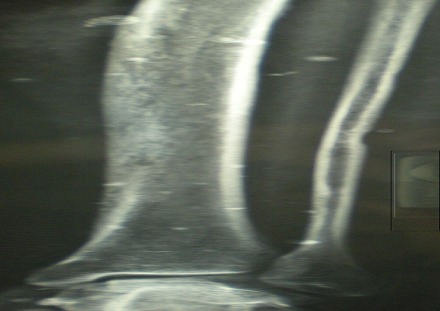
Radiographies standards de la jambe montrant des phlébolithes stigmates d'une malformation veineuses: Vue 3

Il s'agit de Madame B. L., âgée de 27 ans, opérée à l’âge de 3 ans pour un pied bot varus équin gauche, qui consulte dans notre formation pour un syndrome douloureux aigu de la face interne du 1/3 supérieur de la jambe gauche, sans notion de traumatisme, ni fièvre. L'examen clinique a retrouvé une masse palpable de 2 cm très douloureuse à la palpation au niveau de la face interne du 1/3 supérieur de la jambe gauche, mobile par rapport au plan superficiel, adhérente au plan profond. La peau en regard était d'aspect normal. L'examen vasculo-nerveux et les mobilités articulaires du genou et de la cheville homolatéraux étaient normaux. Le bilan radiographique standard avait révélé de multiples calcifications rondes de tailles variables des parties molles de la jambe avec hyperostose tibiale médiale évoquant des phlébolithes (Figure 1, Figure 2, Figure 3).

**Figure 4:**
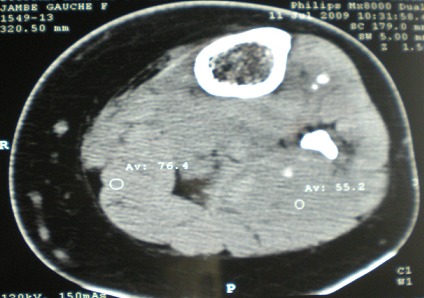
Aspect TDM avec injection du produit de contraste de la MV: Vue 1

**Figure 5:**
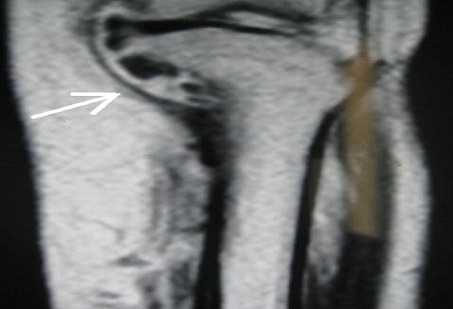
Aspect IRM avec injection du produit de contraste de la MV: Vue 2

Une tomodensitométrie avec injection de produit de contraste a révélé de multiples lésions nodulaires hyperdenses, avec présence d'un nodule isodense au contact de la corticale interne de l'extrémité supérieure du tibia, et un aspect hypervascularisé de la corticale osseuse, s'y associe une petite collection en regard de la corticale osseuse (Figure 4), confirmée par une Imagerie par résonnance magnétique de la jambe gauche (Figure 5). On a procédé sous anesthésie générale à une biopsie-exérèse de la masse antéro-interne du 1/3 supérieur de la jambe gauche, mettant en évidence une masse veineuse, thrombosée avec une calcification centrale. L'examen histologique a révélé une prolifération de lacunes sanguines anfractueuses dont la lumière est très congestive, et limitées par un endothélium atrophique, faisant évoquer un hémangiome caverneux. L’évolution a été marquée par la disparition totale de la douleur.

## Discussion

Les MV sont des anomalies vasculaires à flux lent constituées de veines dysplasiques dont les parois sont déficientes en cellules musculaires lisses [1]. Elles représentent les 2/3 des malformations vasculaires [2]. Ces malformations se voient de manière localisée ou diffuse, superficielle ou profonde, siégeant dans n′importe quelle partie du corps et dans n′importe quel tissu, que ce soit la peau, les muqueuses, les muscles, mais aussi les articulations, les nerfs, les os et les organes internes. Le plus souvent uniques, ces malformations peuvent aussi s′intégrer dans un syndrome plus complexe sporadique ou héréditaire [3].

Dans les MV profondes, la peau est de coloration normale, elle peut rester longtemps méconnue, et n'entraine souvent aucun symptôme avant l’âge de la puberté. La localisation musculaire au niveau des membres semble la plus fréquente [4].

Le diagnostic est suspecté devant: douleurs à l'effort ou après un traumatisme, un épisode douloureux suite à un saignement intralésionnel; rarement, une augmentation de volume entrainant un symptôme compressif [3]; quelquefois, la découverte est fortuite à l'occasion d'une radiographie standard.

Ces MV sont présentes dès la naissance, mais peuvent se manifester plus tard, la MV grandit avec l′enfant et peut s′aggraver inexorablement au fil des années [5]. Son évolution ponctuée par des thromboses localisées responsables d’épisodes douloureux durant une dizaine de jours, à l'origine des phlébolithes, calcifications rondes pathognomoniques de la MV, quelquefois perceptibles à l'examen clinique et toujours visibles à l'examen radiologique. Ces thromboses locales ne sont jamais responsables d'embolie pulmonaire à l'inverse des phlébites du réseau veineux profond [4].

Le traitement des malformations veineuses (MV) est délicat. En effet, les MV sont souvent mal délimitées infiltrant les tissus. Il fait appel à la sclérothérapie ou la chirurgie. La chirurgie d′exérèse radicale ou partielle joue une place prépondérante dans leur traitement lorsqu′elles deviennent symptomatiques [6].

## Conclusion

Étant donné l′absence d'un traitement spécifique ciblé sur leur étiopathogénie encore méconnue, le traitement des MV en est dès lors rarement curatif.
